# Clinical signs and symptoms of Wilson disease in a real-world cohort of patients in the United States: a medical chart review study

**DOI:** 10.3389/fgstr.2023.1299182

**Published:** 2024-01-04

**Authors:** Valentina Medici, Nehemiah Kebede, Jennifer Stephens, Mary Kunjappu, John M. Vierling

**Affiliations:** ^1^ Department of Internal Medicine, Division of Gastroenterology and Hepatology, University of California Davis, Sacramento, CA, United States; ^2^ OPEN Health Evidence and Access, Parsippany, NJ, United States; ^3^ Alexion, AstraZeneca Rare Disease, Boston, MA, United States; ^4^ Departments of Medicine and Surgery, Section of Gastroenterology and Hepatology and Division of Abdominal Transplantation, Baylor College of Medicine, Houston, TX, United States

**Keywords:** Wilson disease, disease signs, disease symptoms, chart review, real world, United States

## Abstract

**Introduction:**

There are limited data from the United States regarding the real-world signs and symptoms of Wilson disease (WD). This retrospective, observational medical chart review was conducted to identify real-world characteristics of patients with WD in the United States, as well as WD signs and symptoms at diagnosis and over time.

**Methods:**

De-identified clinical data were abstracted from medical charts of US patients diagnosed with WD between January 1, 2012, and June 30, 2017. Hepatic, neurologic, and psychiatric biochemical findings, signs, and symptoms were characterized at diagnosis and follow-up/during treatment.

**Results:**

In total, 225 WD patients were included in the study. The mean (SD) age at diagnosis was 24.7 (9.8) years, and 65.3% were male. Median (Q1–Q3) follow-up after diagnosis was 39.5 (33.8–60.4) months. The most common disease presentation at WD diagnosis was combined neurologic/psychiatric and hepatic (52.9%), followed by neurologic/psychiatric (20.0%), hepatic (16.9%), and asymptomatic (10.2%). Common clinical characteristics at diagnosis were Kayser-Fleischer rings (77.2%), low ceruloplasmin levels (95.2%), high hepatic copper (97.8%), elevated 24-hour urinary copper excretion (90.2%), and abnormal liver function tests (38.7%–85.1%). At diagnosis, the most common biochemical findings or hepatic sign/symptoms were abnormal liver enzymes (50.7%), abdominal pain (16.6%), and fatigue (15.7%). The most common neurologic signs/symptoms were headache (18.3%), dysarthria (17.4%), and ataxia (17.0%). Common psychiatric signs/symptoms included anxiety/depression/other mood changes (36.2%), emotional lability (12.8%), and increased irritability/anger outbursts (9.2%). Prevalence of biochemical abnormalities or signs/symptoms among patients at diagnosis and after ~1-year follow-up were neurologic (60.1% and 44.0%), hepatic (69.6% and 37.8%), and psychiatric (53.7% and 37.6%), respectively. Common new onset symptoms at ~1-year post-WD diagnosis were abnormal liver enzymes (5.6%), headache (6.2%), and anxiety/depression/other mood changes (7.2%).

**Conclusions:**

These real-world, descriptive data highlight the clinical complexity and heterogeneity of WD and the need for better education about diagnostic testing and multidisciplinary support. Although rare, the neurologic, psychiatric, and hepatic signs/symptoms of WD have a substantial clinical impact.

## 1 Introduction

Wilson disease (WD) is a genetic disorder caused by inherited mutations in the copper-transporting gene *ATP7B* that is responsible for normal copper homeostasis and biliary excretion from hepatocytes (1). WD results in accumulation of excess copper in the liver, brain, and other tissues, eventually producing a wide variety of hepatic, neurologic, and/or psychiatric signs and symptoms (1). The prevalence of WD in the United States is estimated to range from 1 in 30,000–50,000 people (2). Untreated WD is progressive and can cause death, but timely diagnosis and lifelong adherence to pharmacotherapy can improve abnormal biochemical tests, signs, and symptoms (1, 3, 4).

Because copper accumulation starts in infancy, the clinical signs and symptoms of WD may manifest at any age (5). However, most patients are diagnosed between the ages of 5 and 35 years (4). The clinical presentation of WD with hepatic involvement ranges from asymptomatic elevations of liver enzymes with acute or chronic hepatic histopathology (4) to life-threatening acute liver failure (1). Neurologic disturbances may manifest as difficulty swallowing, drooling, speech disturbances, gait and balance disturbances, movement disorders, muscle rigidity, and tremor (1, 4, 5). In addition, psychiatric manifestations include aggression and irritability, cognitive impairment, emotional lability, mood and/or personality disorders, and psychosis (1).

The diagnosis of WD is complex, since no single diagnostic test unequivocally excludes or confirms WD (4). Therefore, diagnosis requires a detailed history and physical examination, as well as laboratory testing and diagnostic imaging (4). Given the complexity and rarity of WD, patients commonly experience a delayed diagnosis or misdiagnosis (3, 6, 7). In a 2016 survey of patients with confirmed WD (N=97; 93% from the United States), 68% were diagnosed within 1 year of their first symptoms; the remaining 32% of patients experienced diagnostic delays of 1–3 years (18%) or over 3 years (14%) (8). Overall, approximately one third reported being misdiagnosed initially, but 48% of patients with neurologic/psychiatric symptoms reported being misdiagnosed (8). Diagnostic delays have important implications because early treatment initiation is able to prevent complications and improve prognosis (4, 9–11). In addition, the presence of cirrhosis at the time of diagnosis has been associated with worse long-term outcomes (12). Early treatment also helps improve patient quality of life (QoL) (1, 13). In a cross-sectional study of 60 patients with clinically stable WD in Serbia, those with a longer interval between onset of signs and symptoms and of treatment initiation experienced worse QoL (13).

In rare diseases with heterogenous biochemical and clinical manifestations, such as WD, real-world evidence is crucial for understanding the disease’s clinical characteristics and identifying unmet diagnostic and therapeutic needs (14). However, real-world evidence is scarce in US patients with WD. Indeed, a literature search identified only two real-world studies that included US patient data (15). The 2016 multinational qualitative patient survey of 97 patients with WD, mentioned above, assessed clinical presentations, the time interval to accurate diagnosis, and challenges with management (8). A smaller, multinational registry study of 62 patients with WD assessed mental and physical QoL, cognition, and mood, as well as the results of hepatic and neurologic evaluations (15). However, a literature search found no publications about practice patterns of US physicians diagnosing and treating patients with WD. Thus, the present study was conducted to ascertain real-world demographic and clinical characteristics of US patients with WD and to assess WD signs and symptoms at diagnosis and during follow-up.

## 2 Methods

### 2.1 Study design and objectives

**Figure 1:**
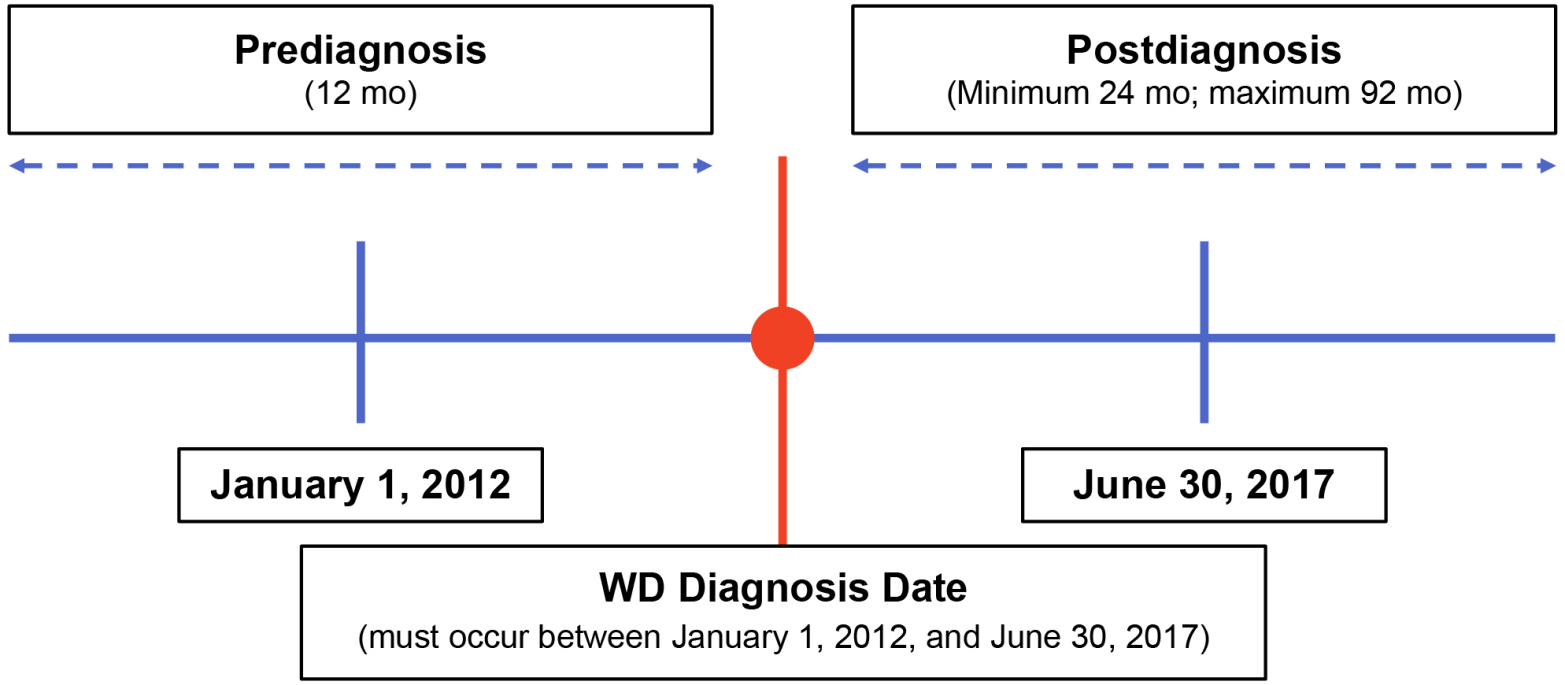
Study design. mo, months; WD, Wilson disease.

This retrospective, observational study used de-identified clinical data abstracted from medical charts of US patients with WD to characterize patient demographics and describe these patients’ clinical signs and symptoms at diagnosis and during follow-up. Physician specialists treating patients with WD were recruited and underwent screening prior to study participation. These physicians were provided specific written instructions for identifying eligible patients for data abstraction. Eligible patients were diagnosed with WD between January 1, 2012, and June 30, 2017 (**Figure 1**). Patients were required to have ≥12 months of clinical data available both prior to and following their WD diagnosis. Clinical data were abstracted from the 12 months pre-diagnosis through their most recent visit or death. Index therapy was defined as any first-line monotherapy or combination therapy with penicillamine, trientine, or zinc. The New England Institutional Review Board, Inc. (Needham, MA) provided ethics approval for this study on December 12, 2019.

### 2.2 Data source and study cohort

Physician specialists (ie, gastroenterologists, hepatologists, neurologists) were initially contacted by email, telephone, and/or facsimile and were screened using a customized questionnaire. Physicians who had treated ≥5 patients with WD initially diagnosed between January 1, 2012, and June 30, 2017, were eligible if they agreed to provide all data points of interest and accepted all study requirements, including data validation and resolution of data queries. Physicians remained anonymous and unaware of the study sponsor. Once qualified, each physician was responsible for identification of eligible patients, data extraction, and completion of patient case report forms. WD patient eligibility requirements included age ≥3 years at time of diagnosis, availability of complete medical records from diagnosis through the most recent visit or death, whichever occurred first. Patients were excluded if they were enrolled in any WD-related clinical trials. Before the study started, details of patient case report forms were vetted by a minimum of 2 participating physicians to ensure reliability and validity.

Key demographic data for physicians included years in practice, medical specialty, and use of published WD treatment guidelines. Key data extracted from patient charts included demographic and clinical/disease characteristics, laboratory tests and signs or symptoms at disease onset (allowing classification as hepatic, neurologic/psychiatric, or both); laboratory tests, signs, symptoms, procedures, and complications at the time of diagnosis; and during follow-up. Unless otherwise stated, all reported follow-up results were obtained at the patients’ most recent follow-up visit.

### 2.3 Statistical analysis

Descriptive statistics were used to describe patient demographics and clinical characteristics. Patient data were de-identified and reported in aggregate. Categorical variables of interest were summarized using the number and percentage of patients in each category. Continuous variables were summarized using mean, standard deviation (SD), median, quartiles (Q), and minimum and maximum values. Results were stratified by initial disease presentation and index monotherapy. The decision to stratify results by index monotherapy was based on the treatment pathway proposed in the European Association for the Study of Liver (EASL) WD clinical guideline (6). Prevalence of symptoms and other clinical manifestations of WD at diagnosis and ~1, 2, and 3 years after diagnosis were also analyzed. Such analyses were limited to patients who had known neurologic, psychiatric, hepatic, or other symptoms at diagnosis and who had a delay of ~1 year from initial assessment and diagnosis.

## 3 Results

### 3.1 Demographics

In total, 44 physicians and 225 of their patients with WD were included in this study. The largest proportion of participating physicians were gastroenterologists (19/44 [43.2%]), followed by hepatologists (15/44 [34.1%]) and neurologists (10/44 [22.7%]). The largest proportion of physicians had been in practice for 6 to 10 years (14/44 [31.8%]) and, at the time of the study, participating physicians were working in academic (23/44 [52.3%]), university-based (25/44 [56.8%]), and/or urban (26/44 [59.1%]) hospital settings. Physicians treated a mean (SD) 11.3 (15.7) patients with WD (median [Q1–Q3], 8.0 [3.0–13.0]). Notably, 40.9% of physicians reported not using WD treatment guidelines in their practice despite caring for ≥5 WD patients.

Patients experienced their first abnormal laboratory test, sign, or symptom at a mean (SD) age of 23.2 (8.9) years (based on data available from 150 patients), while mean (SD) age at the time of diagnosis was 1.5 years older, or 24.7 (9.8) years (data available for all 225 patients) (**Table 1**). The majority of (147/225, 65.3%) patients were male, and 51/225 (22.7%) had a known family history of WD, most commonly a sibling (32/51, 62.7%). Initial treatment was penicillamine monotherapy in 101/225 (44.9%), trientine monotherapy in 58/225 (25.8%), and zinc monotherapy in 13/225 (5.8%) patients. The remaining patients (53/225, 23.6%) initiated treatment with combination therapy.

**Table 1:** Patient demographic characteristics by disease presentation at WD diagnosis^a^.

Variable	Statistic/Category	All Patients (N=225)	Neurologic/ Psychiatric (n=45)	Hepatic (n=38)	Neurologic/ Psychiatric and Hepatic (n=119)	Asymptomatic (n=23)
**Age at first symptom, years**	N	150	37	25	85	3
	Mean (SD)	23.2 (8.9)	21.1 (7.3)	25.1 (12.5)	23.5 (8.3)	23.7 (7.0)
	Median (IQR)	22.0 (16.0–27.0)	22.0 (16.0–24.0)	22.0 (18.0–27.0)	23.0 (17.0–29.0)	23.0 (17.0–31.0)
	Range	8.0–63.0	8.0–45.0	13.0–63.0	9.0–48.0	17.0–31.0
**Age at diagnosis, years**	Mean (SD)	24.7 (9.8)	22.04 (8.27)	27.32 (12.70)	25.50 (9.30)	21.30 (8.67)
**Current age, years**	Mean (SD)	29.1 (9.7)	26.6 (8.2)	31.6 (12.4)	29.8 (9.0)	26.4 (9.2)
**Sex**	Male	147 (65.3)	25 (55.6)	15 (39.5)	87 (73.1)	20 (87.0)
	Female	78 (34.7)	20 (44.4)	23 (60.5)	32 (26.9)	3 (13.0)
**Race**	White	199 (88.4)	41 (91.1)	34 (89.5)	102 (85.7)	22 (95.7)
	Black/African American	13 (5.8)	2 (4.4)	1 (2.6)	10 (8.4)	0 (0.0)
	Asian	9 (4.0)	2 (4.4)	1 (2.6)	6 (5.0)	0 (0.0)
	Native American/Alaska native	2 (0.9)	0 (0.0)	0 (0.0)	1 (0.8)	1 (4.3)
	Multiracial/Unknown	2 (0.9)	0 (0.0)	2 (5.3)	0 (0.0)	0 (0.0)
**Ethnicity**	Not Hispanic or Latino	181 (80.4)	36 (80.0)	28 (73.7)	100 (84.0)	17 (73.9)
	Hispanic or Latino	17 (7.6)	4 (8.9)	2 (5.3)	7 (5.9)	4 (17.4)
	Unknown	27 (12.0)	5 (11.1)	8 (21.1)	12 (10.1)	2 (8.7)
**Family history of WD**	Yes	51 (22.7)	8 (17.8)	17 (44.7)	15 (12.6)	11 (47.8)
	No	139 (61.8)	31 (68.9)	21 (55.3)	77 (64.7)	10 (43.5)
	Unknown	35 (15.6)	6 (13.3)	0 (0.0)	27 (22.7)	2 (8.7)
**Primary major medical insurance**	Commercial	174 (77.3)	37 (82.2)	31 (81.6)	87 (73.1)	19 (82.6)
	Medicaid	48 (21.3)	8 (17.8)	5 (13.2)	31 (26.1)	4 (17.4)
	Medicare	2 (0.9)	0 (0.0)	2 (5.3)	0 (0.0)	0 (0.0)
	Cash/Uninsured	1 (0.4)	0 (0.0)	0 (0.0)	1 (0.8)	0 (0.0)
**Primary pharmacy/drug insurance**	Commercial	174 (77.3)	37 (82.2)	30 (78.9)	88 (73.9)	19 (82.6)
	Medicaid	47 (20.9)	8 (17.8)	5 (13.2)	30 (25.2)	4 (17.4)
	Medicare	3 (1.3)	0 (0.0)	3 (7.9)	0 (0.0)	0 (0.0)
	Cash/Uninsured	1 (0.4)	0 (0.0)	0 (0.0)	1 (0.8)	0 (0.0)
**CCI at diagnosis**	0	177 (78.7)	36 (80.0)	26 (68.4)	93 (78.2)	22 (95.7)
	1	27 (12.0)	8 (17.8)	8 (21.1)	10 (8.4)	1 (4.3)
	≥2	21 (9.3)	1 (2.2)	4 (10.5)	16 (13.4)	0 (0.0)

a Values are n (%) unless otherwise specified. Percentages are of total number of patients with specified disease presentation.

CCI, Charlson Comorbidity Index; IQR, interquartile range; SD, standard deviation; WD, Wilson disease.

### 3.2 Clinical characteristics

Median (Q1–Q3) follow-up after diagnosis was 39.5 (33.8–60.4) months. **Table 2** lists patients’ clinical characteristics and outcomes at the time of WD diagnosis and subsequently during follow-up, stratified according to initial disease presentation as neurologic/psychiatric, hepatic, or both neurologic/psychiatric and hepatic. Signs and symptoms at diagnosis were most often hepatic (157/225, 69.8%), followed by neurologic (135/225, 60.0%), psychiatric (120/225, 53.3%), and other (90/225, 40.0%).

**Table 2:** Patient clinical characteristics and outcomes at the time of WD diagnosis and subsequent follow up.

Statistic/Category		All Patients (N=225)	Neurologic/ Psychiatric (n=45)	Hepatic (n=38)	Neurologic/Psychiatric and Hepatic (n=119)	Asymptomatic (n=23)
**Symptoms at diagnosis, n (%)**	Hepatic	157 (69.8)	0 (0.0)	38 (100.0)	119 (100.0)	0 (0.0)
	Neurologic	136 (60.4)	34 (75.6)	0 (0.0)	102 (85.7)	0 (0.0)
	Psychiatric	121 (53.8)	31 (68.9)	0 (0.0)	90 (75.6)	0 (0.0)
	Other	93 (43.1)	6 (13.3)	10 (26.3)	77 (64.7)	0 (0.0)
	Asymptomatic	23 (10.2)	0 (0.0)	0 (0.0)	0 (0.0)	23 (100.0)
**Fibrosis grade/stage at diagnosis, n (%)**	F0	15 (6.7)	5 (11.1)	1 (2.6)	5 (4.2)	4 (17.4)
	F1	37 (16.4)	4 (8.9)	7 (18.4)	18 (15.1)	8 (34.8)
	F2	55 (24.4)	4 (8.9)	7 (18.4)	41 (34.5)	3 (13.0)
	F3	25 (11.1)	3 (6.7)	6 (15.8)	15 (12.6)	1 (4.3)
	F4	11 (4.9)	0 (0.0)	2 (5.3)	9 (7.6)	0 (0.0)
	Not conducted	82 (36.4)	29 (64.4)	15 (39.5)	31 (26.1)	7 (30.4)
**Eye examination (Kayser–Fleischer rings) at diagnosis, n (%)**	Normal	45 (20.0)	6 (13.3)	7 (18.4)	21 (17.6)	11 (47.8)
	Abnormal	152 (67.6)	32 (71.1)	27 (71.1)	88 (73.9)	5 (21.7)
	Not conducted	28 (12.4)	7 (15.6)	4 (10.5)	10 (8.4)	7 (30.4)
***ATP7B* sequencing test at diagnosis, n (%)**	Positive	124 (55.1)	31 (68.9)	24 (63.2)	58 (48.7)	11 (47.8)
	Not conducted	101 (44.9)	14 (31.1)	14 (36.8)	61 (51.3)	12 (52.2)
**Proportion of patients who received liver transplantation at or since WD diagnosis, n (%)**		18 (8.0)	1 (2.2)	5 (13.2)	12 (10.1)	0 (0.0)
**Proportion of patients who died since last visit, n (%)**		10 (4.4)	4 (8.9)	0 (0.0)	6 (5.0)	0 (0.0)
**Cause of death, n (%)**	WD complications	4 (40.0)	0 (0.0)	0 (0.0)	4 (66.7)	0 (0.0)
	Other	6 (60.0)	4 (100.0)	0 (0.0)	2 (33.3)	0 (0.0)
**Months of follow-up since diagnosis**	Mean (SD)	46.8 (20.2)	46.3 (20.3)	47.4 (20.2)	45.6 (18.7)	53.00 (26.3)
	Median (IQR)	39.5 (33.8–60.4)	37.8 (34.3–59.9)	38.0 (33.2–64.3)	39.8 (33.9–60.1)	46.8 (29.1–82.4)

IQR, interquartile range; SD, standard deviation; WD, Wilson disease.

Fibrosis on liver biopsy was staged in 143/225 (63.6%) patients at WD diagnosis. Among the 143 patients with liver biopsies, 55 (38.5%) presented with fibrosis stage F2, 25 (17.5%) presented with F3, and 11 (7.7%) had F4 (cirrhosis). Kayser–Fleischer corneal rings were assessed in 197/225 (87.6%) patients at diagnosis and detected in 152/197 (77.2%). Examination for Kayser-Fleischer rings was not performed in 28/225 (12.4%) patients. Testing for *ATP7B* sequencing was performed in only 124/225 (55.1%) of patients, and 100% patients had mutations. Conversely, *ATP7B* sequencing was not performed in 101/225 (44.9%) patients. A small proportion of patients (18/225, 8.0%) underwent liver transplantation at either the time of WD diagnosis or thereafter. Among all patients with WD, 10/225 (4.4%) died during follow-up, and 4/10 (40.0%) of deaths were attributed to WD complications.

**Figure 2:**
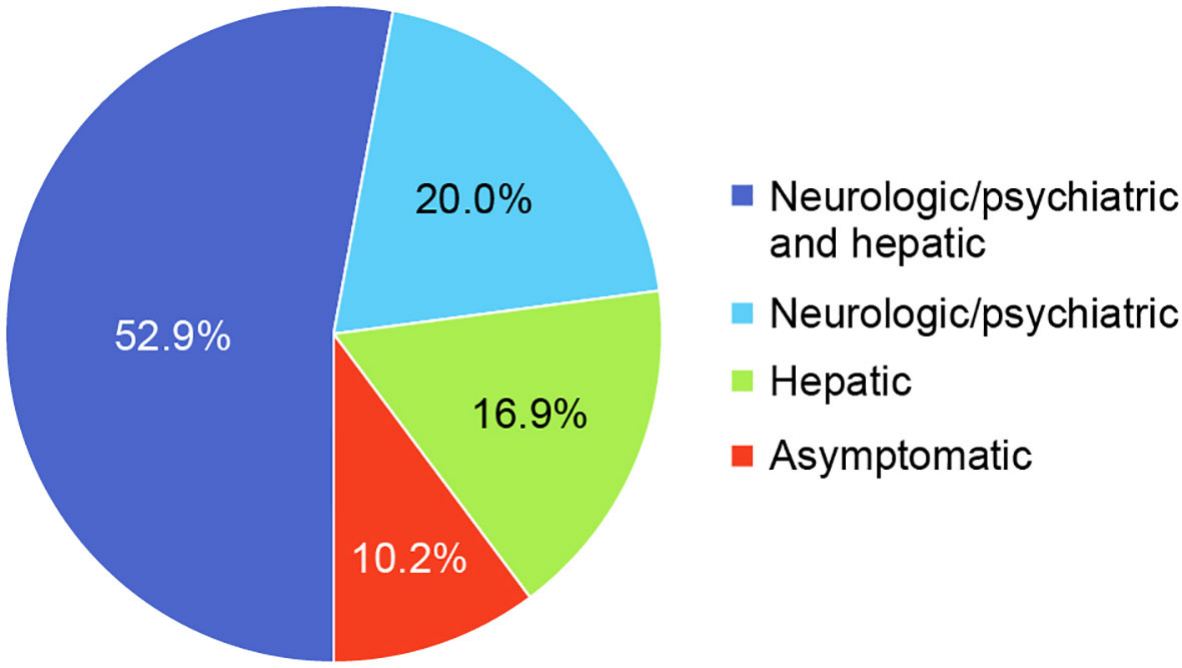
Disease presentation at diagnosis.

The most common clinical manifestations of WD at the time of diagnosis involved combined neurologic/psychiatric and hepatic (119/225, 52.9%), followed by neurologic/psychiatric (45/225, 20.0%), hepatic (38/225, 16.9%), and asymptomatic (23/225, 10.2%) (**Figure 2**). Among patients with hepatic presentations, 23/38 (60.5%) were females, while males predominated among patients classified as neurologic/psychiatric (25/45 [55.6%]), combined neurologic/psychiatric and hepatic (87/119 [73.1%]), or asymptomatic (20/23 [87.0%]).

**Figure 3:**
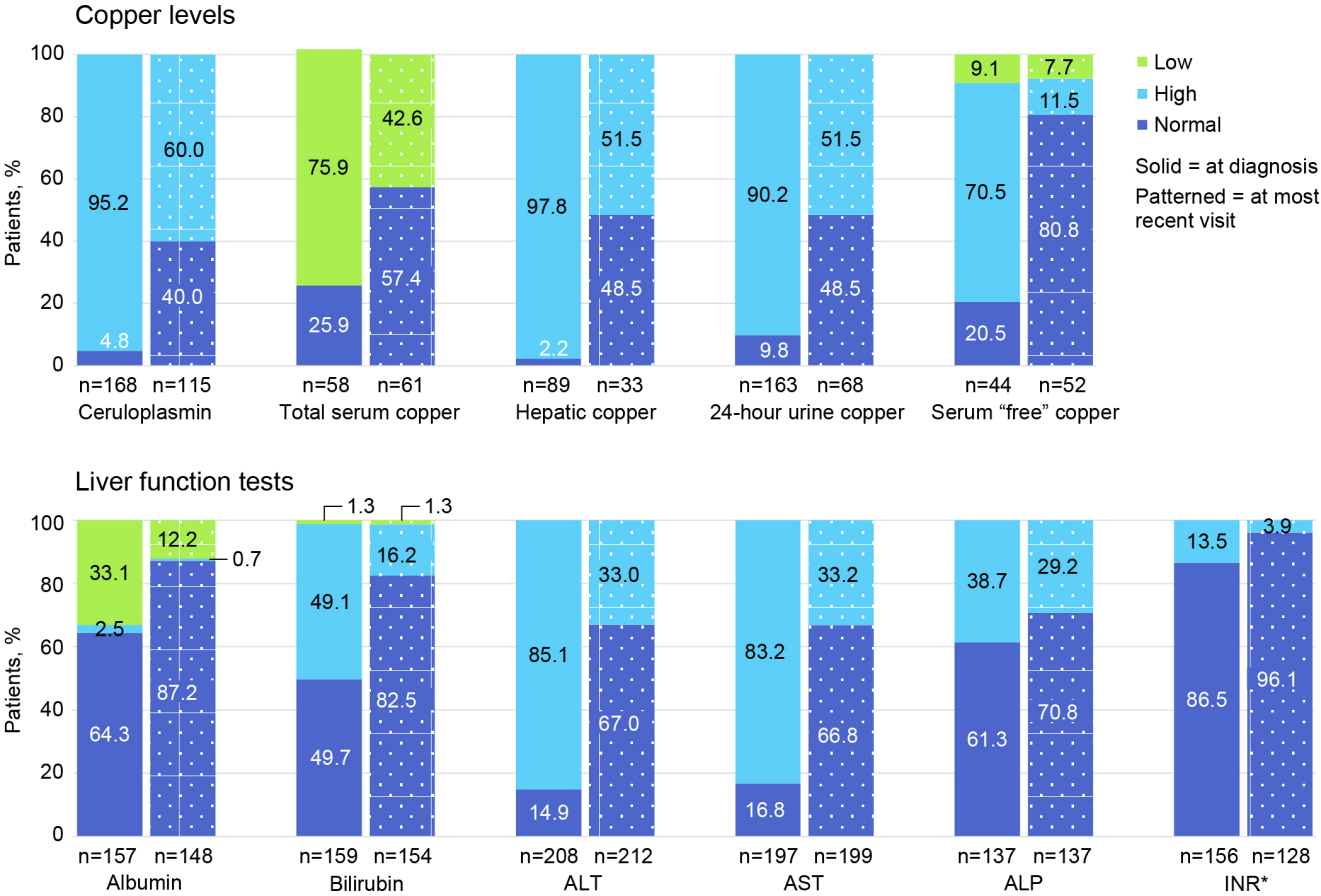
Proportion of patients with low, high, and normal laboratory values at diagnosis and at most recent follow-up visit. ALP, alkaline phosphatase; ALT, alanine aminotransferase; AST, aspartate aminotransferase; INR, international normalized ratio. *For INR, normal is ≤2.0 and high is >2.0.

**Figure 3** shows the proportion of patients with low, high, and normal laboratory values at diagnosis and at most recent follow-up visit. Only 168/225 (74.7%) had plasma ceruloplasmin measured at WD diagnosis, and only 115/225 (51.1%) had ceruloplasmin testing at last follow-up. A low ceruloplasmin was found in 160/168 (95.2%) patients tested at WD diagnosis and in 69/115 (60.0%) tested at last follow-up. At the time of WD diagnosis, only 163/225 (72.4%) patients had ≥1 laboratory test for copper (ie, total serum copper, hepatic copper quantification, 24-hour urinary copper excretion, and/or “free” or non-ceruloplasmin bound serum copper). In contrast, testing for copper using ≥1 of these tests at last follow-up occurred in only 68/225 (30.2%) patients. At diagnosis, 44/58 (75.9%) patients had a low total serum copper concentration; 87/89 (97.8%) had elevated quantitative hepatic copper; 147/163 (90.2%) had elevated 24-hour urinary copper excretion; and 31/44 (70.5%) had elevated serum “free” or non-ceruloplasmin bound copper. At last follow-up, 26/61 (42.6%) had low total serum copper; 17/33 (51.5%) had elevated hepatic copper; 35/68 (51.5%) had elevated 24-hour urinary copper; and 6/52 (11.5%) had elevated serum “free” or non-ceruloplasmin bound copper.

At diagnosis, 208 of 225 (92.4%) patients had ≥1 LFT (ie, alanine aminotransferase [ALT], aspartate aminotransferase [AST], and alkaline phosphatase [ALP]); at follow-up, 212 of 225 (94.2%) had ≥1 LFT. Specifically, at diagnosis, 177 of 208 (85.1%) had elevated ALT; 164 of 197 (83.2%) had elevated AST; and 53 of 137 (38.7%) had elevated ALP levels. At follow-up, 70 of 212 (33.0%) had elevated ALT; 66 of 199 (33.2%) had elevated AST; and 40 of 137 (29.2%) had ALP levels.

Imaging studies were performed in a minority of patients at the time of WD diagnosis: brain magnetic resonance imaging (MRI) in 59 of 225 (26.2%) patients and liver (MRI or computed tomography [CT]) in 47 of 225 (20.9%). Brain MRI was performed at last follow-up in 29 of 225 (12.9%) and abdominal MRI or CT scan in 25 of 225 (11.1%) patients. Brain MRI was abnormal in 15/59 (25.4%) patients at diagnosis and in 3/29 (10.3%) at follow-up. Abdominal MRI or CT scans were abnormal in 31/47 (66.0%) patients at diagnosis and 15/25 (60.0%) at follow-up.

### 3.3 Prevalence of clinical signs and symptoms

**Table 3** lists the prevalence of clinical signs and symptoms at diagnosis and at ~1 year after diagnosis for all patients, stratified according to index monotherapy. A total of 151/217 (69.6%) patients had ≥1 hepatic sign or symptom at diagnosis with a median of 1 symptom per patient; after ~1 year of follow-up, this proportion decreased to 82/217 (37.8%). A total of 131/218 (60.1%) patients had ≥1 neurologic sign or symptom at diagnosis, with a median of 2 symptoms per patient; after ~1 year of follow-up, this proportion decreased to 96/218 (44.0%). Finally, 117/218 (53.7%) patients had ≥1 psychiatric sign or symptom at diagnosis, with a median of 1 symptom per patient; after ~1 year of follow-up, this proportion decreased to 82/218 (37.6%).

**Table 3:** Period prevalence of symptoms at diagnosis and during follow-up.

Variable	Statistic/ Category	All Patients		Penicillamine Monotherapy		Trientine Monotherapy		Zinc Monotherapy	
		At Diagnosis	~1 Year After Diagnosis	At Diagnosis	~1 Year After Diagnosis	At Diagnosis	~1 Year After Diagnosis	At Diagnosis	~1 Year After Diagnosis
**Proportion of patients with ≥1** **known hepatic symptom, n (%)**		151 (69.6)	82 (37.8)	80 (82.5)	43 (44.3)	37 (67.3)	16 (29.1)	9 (69.2)	2 (15.4)
**Total number of hepatic symptoms among patients with ≥1 hepatic symptom**	Mean (SD)	1.95 (1.25)	2.06 (1.49)	1.89 (1.29)	2.26 (1.51)	2.08 (1.09)	1.69 (0.79)	2.67 (1.94)	6.00 (1.41)
	Median (Q1–Q3)	1.0 (1.0–3.0)	1.5 (1.0–2.0)	1.0 (1.0–3.0)	2.0 (1.0–3.0)	2.0 (1.0–3.0)	2.0 (1.0–2.0)	2.0 (1.0–4.0)	6.0 (5.0–7.0)
	Range	1.0–7.0	1.0–7.0	1.0–7.0	1.0–7.0	1.0–6.0	1.0–4.0	1.0–6.0	5.0–7.0
**Proportion of patients with ≥1** **known neurologic symptom, n (%)**		131 (60.1)	96 (44.0)	53 (54.6)	41 (42.3)	35 (62.5)	19 (33.9)	9 (69.2)	3 (23.1)
**Total number of neurologic symptoms among patients with ≥1 neurologic symptom**	Mean (SD)	2.31 (1.31)	2.41 (1.66)	2.32 (1.22)	2.54 (1.47)	1.94 (1.00)	1.58 (0.90)	2.67 (1.58)	2.00 (1.73)
	Median (Q1–Q3)	2.0 (1.0–3.0)	2.0 (1.0–3.0)	2.0 (1.0–3.0)	2.0 (1.0–3.0)	2.0 (1.0–3.0)	1.0 (1.0–2.0)	3.0 (1.0–3.0)	1.0 (1.0–4.0)
	Range	1.0–8.0	1.0–11.0	1.0–6.0	1.0–6.0	1.0–5.0	1.0–4.0	1.0–5.0	1.0–4.0
**Proportion of patients with ≥1** **known psychiatric symptom, n (%)**		117 (53.7)	82 (37.6)	52 (53.6)	36 (37.1)	32 (57.1)	16 (28.6)	7 (53.8)	2 (15.4)
**Total number of psychiatric symptoms among patients with ≥1 psychiatric symptom**	Mean (SD)	1.61 (0.91)	1.72 (1.05)	1.65 (0.88)	1.83 (0.97)	1.44 (0.80)	1.38 (0.62)	1.86 (0.90)	2.00 (0.00)
	Median (Q1–Q3)	1.0 (1.0–2.0)	1.0 (1.0–2.0)	1.0 (1.0–2.0)	2.0 (1.0–2.0)	1.0 (1.0–2.0)	1.0 (1.0–2.0)	2.0 (1.0–3.0)	2.0 (2.0–2.0)
	Range	1.0–5.0	1.0–6.0	1.0–4.0	1.0–4.0	1.0–5.0	1.0–3.0	1.0–3.0	2.0–2.0

Q, quartile; SD, standard deviation.

**Figure 4:**
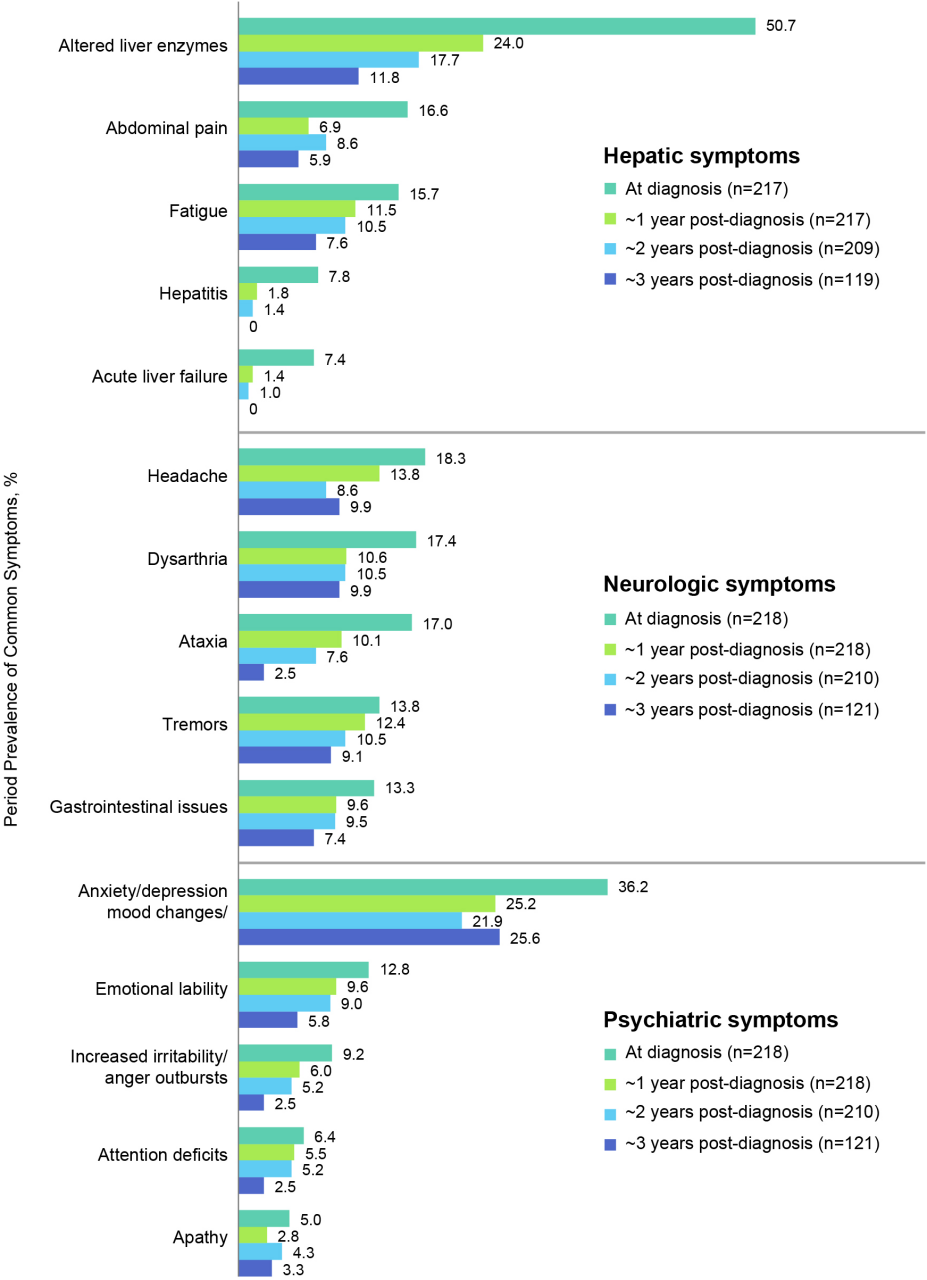
Period prevalence of most common hepatic, neurologic, and psychiatric symptoms at diagnosis and at 1, 2, and 3-year follow-up.

**Figure 4** shows the most prevalent hepatic, neurologic, and psychiatric signs, symptoms, and findings at diagnosis and their prevalences at ~1, 2, and 3 years after diagnosis (see also **Table 4** for a list of all prevalent signs, symptoms, and findings). The most common hepatic signs, symptoms, and findings at WD diagnosis included abnormal liver enzymes (110/217, 50.7%), abdominal pain (36/217, 16.6%), and fatigue (34/217, 15.7%). The prevalence at ~1 year for abnormal liver enzymes was 52/217 (24.0%), for abdominal pain was 15/217 (6.9%), and for fatigue was 25/217 (11.5%). The most common neurologic symptoms were headache (40/218, 18.3%), dysarthria/slurred speech/speech disturbances (38/218, 17.4%), and ataxia (37/218, 17.0%). The prevalence at ~1 year for headache was 30/218 (13.8%), for dysarthria/slurred speech/speech disturbances was 23/218 (10.6%), and for ataxia was 22/218 (10.1%). The most common psychiatric symptoms at WD diagnosis were anxiety/depression/other mood changes (79/218, 36.2%), emotional lability (28/218, 12.8%), and increased irritability/anger outbursts (20/218, 9.2%). The prevalence at ~1 year for anxiety/depression/other mood changes was 55/218 (25.2%), for emotional lability was 21/218 (9.6%), and for increased irritability/anger outbursts was 13/218 (6.0%). Notably, none of these common signs, symptoms, or findings was more prevalent at ~1 year than at diagnosis.

**Table 4:** Period prevalence of all symptoms at diagnosis and at 1-year follow-up, by index monotherapy.

Period Prevalence of Symptoms, n (%)	All Patients		Penicillamine Monotherapy		Trientine Monotherapy		Zinc Monotherapy	
	At Diagnosis	~1 Year After Diagnosis	At Diagnosis	~1 Year After Diagnosis	At Diagnosis	~1 Year After Diagnosis	At Diagnosis	~1 Year After Diagnosis
Patients assessed for neurologic signs and symptoms	N=218		n=97		n=56		n=13	
Ataxia	37 (17.0)	22 (10.1)	20 (20.6)	13 (13.4)	5 (8.9)	0 (0.0)	4 (30.8)	0 (0.0)
Dysarthria/slurred speech/speech disturbances	38 (17.4)	23 (10.6)	16 (16.5)	10 (10.3)	8 (14.3)	2 (3.6)	4 (30.8)	1 (7.7)
Dysexecutive syndrome	4 (1.8)	2 (0.9)	2 (2.1)	0 (0.0)	1 (1.8)	0 (0.0)	0 (0.0)	0 (0.0)
Dyskinesia/dystonia	10 (4.6)	16 (7.3)	4 (4.1)	7 (7.2)	3 (5.4)	3 (5.4)	0 (0.0)	0 (0.0)
Dysphagia/difficulty swallowing	13 (6.0)	13 (6.0)	6 (6.2)	7 (7.2)	3 (5.4)	3 (5.4)	1 (7.7)	0 (0.0)
Gait abnormalities	19 (8.7)	16 (7.3)	9 (9.3)	9 (9.3)	0 (0.0)	1 (1.8)	2 (15.4)	1 (7.7)
GI issues (constipation, nausea/vomiting, other)	29 (13.3)	21 (9.6)	14 (14.4)	10 (10.3)	11 (19.6)	5 (8.9)	0 (0.0)	0 (0.0)
Handwriting issues	17 (7.8)	11 (5.0)	6 (6.2)	4 (4.1)	7 (12.5)	2 (3.6)	1 (7.7)	1 (7.7)
Headache	40 (18.3)	30 (13.8)	18 (18.6)	16 (16.5)	13 (23.2)	5 (8.9)	5 (38.5)	2 (15.4)
Impaired bladder control	1 (0.5)	0 (0.0)	0 (0.0)	0 (0.0)	1 (1.8)	0 (0.0)	0 (0.0)	0 (0.0)
Increased salivation, drooling	3 (1.4)	3 (1.4)	1 (1.0)	2 (2.1)	0 (0.0)	0 (0.0)	1 (7.7)	0 (0.0)
Muscle rigidity (stiffness)	13 (6.0)	11 (5.0)	3 (3.1)	5 (5.2)	2 (3.6)	2 (3.6)	1 (7.7)	0 (0.0)
Parkinsonism	14 (6.4)	15 (6.9)	3 (3.1)	8 (8.2)	2 (3.6)	0 (0.0)	1 (7.7)	0 (0.0)
Restless legs	8 (3.7)	9 (4.1)	3 (3.1)	5 (5.2)	2 (3.6)	0 (0.0)	0 (0.0)	0 (0.0)
Seizures	5 (2.3)	3 (1.4)	2 (2.1)	1 (1.0)	1 (1.8)	0 (0.0)	0 (0.0)	0 (0.0)
Sleep disturbance	20 (9.2)	8 (3.7)	9 (9.3)	3 (3.1)	5 (8.9)	3 (5.4)	2 (15.4)	0 (0.0)
Syncope/fainting	2 (0.9)	1 (0.5)	1 (1.0)	0 (0.0)	0 (0.0)	0 (0.0)	0 (0.0)	0 (0.0)
Tremors	30 (13.8)	27 (12.4)	6 (6.2)	4 (4.1)	4 (7.1)	4 (7.1)	2 (15.4)	1 (7.7)
Patients assessed for psychiatric symptoms	N=218		n=97		n=56		n=13	
Anxiety, depression, or other mood changes	79 (36.2)	55 (25.2)	34 (35.1)	24 (24.7)	23 (41.1)	10 (17.9)	5 (38.5)	2 (15.4)
Apathy	11 (5.0)	6 (2.8)	9 (9.3)	4 (4.1)	1 (1.8)	1 (1.8)	0 (0.0)	0 (0.0)
Attention deficits	14 (6.4)	12 (5.5)	5 (5.2)	8 (8.2)	4 (7.1)	1 (1.8)	1 (7.7)	0 (0.0)
Bipolar disorder/mania	6 (2.8)	5 (2.3)	3 (3.1)	2 (2.1)	2 (3.6)	2 (3.6)	0 (0.0)	0 (0.0)
Cognitive impairment	8 (3.7)	9 (4.1)	5 (5.2)	3 (3.1)	0 (0.0)	0 (0.0)	0 (0.0)	0 (0.0)
Disinhibition	5 (2.3)	4 (1.8)	1 (1.0)	2 (2.1)	3 (5.4)	0 (0.0)	1 (7.7)	0 (0.0)
Emotional lability	28 (12.8)	21 (9.6)	14 (14.4)	11 (11.3)	6 (10.7)	5 (8.9)	2 (15.4)	1 (7.7)
Hyperactivity	8 (3.7)	8 (3.7)	2 (2.1)	2 (2.1)	2 (3.6)	3 (5.4)	1 (7.7)	0 (0.0)
Increased irritability/anger outbursts	20 (9.2)	13 (6.0)	9 (9.3)	7 (7.2)	5 (8.9)	0 (0.0)	3 (23.1)	1 (7.7)
Psychosis	7 (3.2)	6 (2.8)	3 (3.1)	3 (3.1)	0 (0.0)	0 (0.0)	0 (0.0)	0 (0.0)
Self-injurious behavior	2 (0.9)	2 (0.9)	1 (1.0)	0 (0.0)	0 (0.0)	0 (0.0)	0 (0.0)	0 (0.0)
Patients assessed for hepatic signs and symptoms	N=217		n=97		n=55		n=13	
Abdominal pain	36 (16.6)	15 (6.9)	21 (21.6)	12 (12.4)	11 (20.0)	1 (1.8)	2 (15.4)	0 (0.0)
Acute liver failure	16 (7.4)	3 (1.4)	8 (8.2)	2 (2.1)	2 (3.6)	0 (0.0)	4 (30.8)	1 (7.7)
Altered liver enzymes	110 (50.7)	52 (24.0)	58 (59.8)	28 (28.9)	28 (50.9)	8 (14.5)	6 (46.2)	2 (15.4)
Ascites/swelling in abdomen	7 (3.2)	9 (4.1)	2 (2.1)	6 (6.2)	1 (1.8)	0 (0.0)	1 (7.7)	2 (15.4)
Cirrhosis	10 (4.6)	9 (4.1)	5 (5.2)	4 (4.1)	1 (1.8)	1 (1.8)	1 (7.7)	1 (7.7)
Edema/swelling in lower limbs	8 (3.7)	10 (4.6)	5 (5.2)	5 (5.2)	2 (3.6)	3 (5.5)	1 (7.7)	1 (7.7)
Fatigue	34 (15.7)	25 (11.5)	15 (15.5)	12 (12.4)	12 (21.8)	10 (18.2)	2 (15.4)	1 (7.7)
Fatty liver	11 (5.1)	4 (1.8)	5 (5.2)	4 (4.1)	4 (7.3)	0 (0.0)	1 (7.7)	0 (0.0)
Frailty	1 (0.5)	0 (0.0)	1 (1.0)	0 (0.0)	0 (0.0)	0 (0.0)	0 (0.0)	0 (0.0)
Gastroesophageal varices with or without hemorrhage	3 (1.4)	3 (1.4)	2 (2.1)	2 (2.1)	1 (1.8)	0 (0.0)	0 (0.0)	0 (0.0)
Hepatic encephalopathy	8 (3.7)	2 (0.9)	5 (5.2)	2 (2.1)	0 (0.0)	0 (0.0)	2 (15.4)	0 (0.0)
Hepatitis	17 (7.8)	4 (1.8)	6 (6.2)	3 (3.1)	7 (12.7)	0 (0.0)	1 (7.7)	1 (7.7)
Hepatomegaly	4 (1.8)	4 (1.8)	2 (2.1)	2 (2.1)	1 (1.8)	1 (1.8)	0 (0.0)	0 (0.0)
Hyperammonemia	3 (1.4)	2 (0.9)	0 (0.0)	1 (1.0)	0 (0.0)	0 (0.0)	1 (7.7)	0 (0.0)
Jaundice/yellow skin	10 (4.6)	7 (3.2)	5 (5.2)	3 (3.1)	2 (3.6)	1 (1.8)	2 (15.4)	2 (15.4)
Liver transplantation	0 (0.0)	2 (0.9)	0 (0.0)	0 (0.0)	0 (0.0)	0 (0.0)	0 (0.0)	1 (7.7)
Pleural effusion	0 (0.0)	1 (0.5)	0 (0.0)	1 (1.0)	0 (0.0)	0 (0.0)	0 (0.0)	0 (0.0)
Splenomegaly	2 (0.9)	4 (1.8)	1 (1.0)	3 (3.1)	1 (1.8)	1 (1.8)	0 (0.0)	0 (0.0)
Tendency to bleed easily	2 (0.9)	2 (0.9)	2 (2.1)	2 (2.1)	0 (0.0)	0 (0.0)	0 (0.0)	0 (0.0)
Thrombocytopenia	9 (4.1)	5 (2.3)	6 (6.2)	3 (3.1)	3 (5.5)	1 (1.8)	0 (0.0)	0 (0.0)
Vomiting	3 (1.4)	6 (2.8)	2 (2.1)	2 (2.1)	1 (1.8)	0 (0.0)	0 (0.0)	0 (0.0)

GI, gastrointestinal.

Among all patients with known hepatic symptoms at WD diagnosis and at ≥1 time point during follow-up (n=217), the most common new onset hepatic findings at ~1 year after WD diagnosis were abnormal liver enzymes (6/107, 5.6%), fatigue (9/183, 4.9%), and ascites/swelling in abdomen (6/210, 2.9%). For patients treated initially with penicillamine monotherapy (n=97), the most common new onset hepatic findings at ~1 year after diagnosis were abnormal liver enzymes (4/39, 10.3%), ascites/swelling in abdomen (5/95, 5.3%), and fatigue (4/82, 4.9%). For patients treated initially with trientine monotherapy (n=55), the most common new onset hepatic findings at ~1 year after diagnosis were fatigue (2/43, 4.6%), edema/swelling in lower limbs (2/53, 3.8%), and abnormal liver enzymes (1/27, 3.7%). For patients treated initially with zinc monotherapy (n=13), the most common new onset hepatic findings at ~1 year after diagnosis were abnormal liver enzymes (1/7, 14.3%), fatigue (1/11, 9.1%), edema/swelling in lower limbs (1/12, 8.3%), and ascites/swelling in abdomen (1/12, 8.3%).

Among all patients with known neurologic symptoms at WD diagnosis and at ≥1 time point during follow-up (n=218), the most common new onset neurologic findings at 1 year after WD diagnosis included headache (11/178, 6.2%), ataxia (9/181, 5.0%), and dysarthria/slurred speech/speech disturbances (8/180, 4.4%). For patients treated initially with penicillamine monotherapy (n=97), the most common new onset neurologic findings at ~1 year after WD diagnosis were headache (6/79, 7.6%), Parkinsonism (6/94, 6.4%), and muscle rigidity (5/94, 5.3%). For patients treated initially with trientine monotherapy (n=44), the most common new onset neurologic findings at ~1 year after WD diagnosis were sleep disturbance (1/51, 2.0%), tremors (1/52, 1.9%), dyskinesia/dystonia (1/53, 1.9%), and dysphagia (1/53, 1.9%). For patients treated initially with zinc monotherapy (n=13), the only new onset neurologic finding at ~1 year after WD diagnosis was gait abnormalities (1/11, 9.1%).

Among all patients with known psychiatric symptoms at WD diagnosis and at ≥1 time point during follow-up (n=218), the most common new onset psychiatric symptoms at ~1 year after WD diagnosis included anxiety/depression/other mood changes (10/139, 7.2%), emotional lability (8/190, 4.2%), and increased irritability/anger outbursts (7/198, 3.5%). For patients treated initially with penicillamine monotherapy (n=97), the most common new onset psychiatric symptoms at ~1 year after WD diagnosis were anxiety/depression/other mood changes (5/63, 7.9%), emotional lability (6/83, 7.2%), and attention deficits (4/92, 4.3%). Common new onset psychiatric symptoms at ~1 year after WD diagnosis for patients treated initially with trientine monotherapy (n=56) were attention deficits (1/52, 1.9%) and hyperactivity (1/54, 1.8%). There were no new onset psychiatric symptoms after ~1 year reported for patients treated initially with zinc monotherapy (n=13).

## 4 Discussion

This observational, retrospective, real-world study describes the demographic and clinical characteristics of 225 US patients with WD who had been diagnosed by clinicians selected on the basis of their experience in the diagnosis and treatment of WD (i.e., caring for ≥5 patients with WD within <10 years). Medical records of each patient were reviewed to identify the prevalence of biochemical abnormalities, signs, and symptoms of WD at the time of diagnosis and the incidence of new onset abnormalities after ~1 year of follow-up. The most common clinical classifications of presentations of WD at diagnosis were combined neurologic/psychiatric and hepatic; reflecting the high rate of abnormal liver enzymes and neurologic/psychiatric signs or symptoms at diagnosis. This mixed presentation may contribute to a diagnostic bias favoring evaluation for WD in patients presenting with a combination of hepatic, neurologic, and psychiatric abnormalities. In the present study, one fifth of physicians were neurologists with personal experience of treating patients with WD, which may have increased their index of suspicion for the neurologic and psychiatric manifestations of WD. The neurologic and psychiatric signs and symptoms at presentation would also be detectable from a history and complete physical examination by non-neurologists, while abnormal liver enzymes would have directed attention to the possibility of WD. The fact that most patients presented with a combination of neurologic/psychiatric and hepatic abnormalities of WD highlights the need for multidisciplinary training in the diagnosis and management of WD.

The mixed neurologic, psychiatric, and hepatic presentation observed in this study also challenges the entrenched belief that hepatic presentations, either asymptomatic or symptomatic, predominate in WD. The predominance of hepatic presentations reported in previous cohort studies may reflect differences in diagnostic approaches and clinical practice settings (7, 12, 16). For example, in Austria, a retrospective study of patients diagnosed with WD between 1961 and 2013 (N=229) found that 61.1% had hepatic disease and 26.6% had neurologic disease at presentation (12). However, in the Austrian study, hepatic copper content was measured in all patients with biopsy specimens, regardless of liver biopsy indication (12). This practice undoubtedly increased the potential to identify hepatic WD (12). In Portugal, a retrospective evaluation of patients diagnosed with WD (N=24) between 1975 and 2020 identified hepatic disease in 70.8% and neurologic disease in 25.0% at diagnosis (16). Similarly, in Germany, a cohort study of patients with a WD diagnosis established between 2000 and 2005 (N=163) found that 58.9% had hepatic disease and 33.7% had neurologic disease at diagnosis (7). Not surprisingly, many patients in these studies were diagnosed within a hepatology department or primarily by gastroenterologists/hepatologists (7, 12, 16), which could reflect a referral bias favoring identification of patients with hepatic rather than neurologic disease.

In the absence of universally established standards for classifying or categorizing WD disease patient presentation, it is impossible to assess the frequency of mixed neurologic, psychiatric, and hepatic findings at the time of WD diagnosis. The presentations of the Portuguese and German patients described above were categorized as asymptomatic, hepatic, neurologic, and/or ophthalmologic disease (7, 16). In Greece, a 2020 retrospective chart review that included patients with WD diagnosed over the preceding 30 years (N=63) also identified more patients with hepatic disease (76.2%) than with neurologic disease (20.6%) (17). These patients were described as presenting with hepatic, neurologic, or “other” disease (17). One potential solution would be development of a multispecialty application checklist for practitioners to use while assessing individual patients.

The present analysis also identified problematic diagnostic approaches to WD and key diagnostic findings in the United States, where only 40.9% of physicians caring for ≥5 WD patients disappointingly reported using published practice guidelines in their practices. Thus, it is not surprising that their diagnostic approaches differed from the recommendations of the American Association for the Study of Liver Diseases (AASLD) guidance recommendations for the diagnosis and management of WD (11). The AASLD guidance, recently updated in 2022, describes 2 diagnostic approaches for patients with suspected WD: one based on presence of hepatic disease, and the other based on presence of neurologic disease (11). Briefly, in both approaches, baseline testing to identify Kayser–Fleischer rings (using slit-lamp examination or optical tomography), low serum ceruloplasmin (lower limit of normal, 20 mg/dL), and high basal 24-hour urinary copper (upper limit of normal, 40 µg/24h) (11). If the results of any of these tests are inconclusive, liver biopsy for histology and quantification of hepatic copper concentration and genetic testing for *ATP7B* mutations are warranted (11). In addition, patients with neurologic abnormalities should undergo MRI of the brain (11).

In the current study, the most commonly used diagnostic test was an corneal examination for Kayser–Fleischer rings (87.6%), which was positive in a majority of patients (77.2%). Surprisingly, the best known diagnostic test, plasma ceruloplasmin, was only tested in 74.7% of patients. As expected, a low level was detected in almost all (95.2%) of the patients tested. The 24-hour urinary copper excretion test was the most common laboratory test performed for copper metabolism (72.4%), and 90.2% had abnormally high urinary copper levels. Total serum copper was infrequently measured (19.5%) at diagnosis, and most patients had abnormally low levels (75.9%). Almost all patients (92.4%) had ≥1 liver biochemical tests at diagnosis, and 38.7%–85.1% had abnormal results. *ATP7B* gene sequencing was performed in 55.1% of patients, and all patients tested had positive results. A liver biopsy to assess hepatic fibrosis was performed in 63.6%, and stage 4 cirrhosis was identified in 7.7%.

In the present study, multiple signs and symptoms and abnormal liver enzyme results were present at diagnosis and after ~1-year follow-up. This testifies to the heterogeneity of presenting features of WD and the substantial disease burden experienced by patients. It also indicates that patients with WD may present to a variety of healthcare providers seeking an accurate diagnosis of a rare disease. In this study, there was 1.5 years between mean age at symptom onset and mean age at diagnosis. At diagnosis, the most common hepatic signs and symptoms included abnormal liver enzymes, abdominal pain, fatigue, hepatitis, and rarely, acute liver failure. Common neurologic signs and symptoms in this patient cohort were headache, dysarthria, ataxia, tremors, and gastrointestinal issues. Common psychiatric symptoms were anxiety/depression/mood changes, emotional lability, increased irritability, attention deficits, and apathy. After ~1 year of follow up, neurologic, psychiatric, and/or hepatic liver enzyme abnormalities and symptoms persisted in 37.6%–44.0% of patients. The prevalence of all the most common symptoms decreased between diagnosis and 1-year follow-up. Incident symptoms occurred in up to 7.9% of patients. This may indicate issues with access to therapies, adherence, or inadequate decopperization with the selected therapies.

Our finding of a wide range of signs, symptoms, and abnormal liver enzymes at the time of diagnosis of WD is in accord with other reports. A 2021 cross-sectional analysis evaluated data collected via an international registry (N=62) that included US patients (15). Although the study did not comprehensively identify WD signs and symptoms, some results are notable. For example, 37.3% of patients had major depressive disorder, 47.5% had cognitive impairment, 80.7% had neurologic symptoms, and 19.7% had cirrhosis (15). These patients were not newly diagnosed and had extensive experience with WD treatment. Indeed, median age at time of registry enrollment was 41 years, median age at diagnosis was 19 years, and median treatment duration was 19.5 years (15). Such data illustrate the durability of some WD symptoms despite ongoing treatment.

A 2021 qualitative study characterized patient experience with WD in a series of semistructured patient interviews (18). A total of 11 patients (8/10 US patients) with WD (63.6% with >11 years since initial diagnosis), all receiving treatment, reported an average of 21 signs and symptoms during the study (18). These interviews informed a conceptual model of patients’ WD experience that included as many as 54 symptoms (22 hepatic, 19 neurologic, 13 psychiatric) (18). Patients also reported that their signs and symptoms had an adverse impact on their lives. Average rating of this adverse impact was ≥7 (10 = most bothersome) for almost one half (49%) of the symptoms experienced (18).

### 4.1 Strengths and limitations

This is the first real-world US study to characterize the clinical signs and symptoms of patients with WD using data abstracted directly from patient charts. This analysis reflects the real-world characteristics of US patients with WD diagnosed and managed by clinicians with verified experience in WD. Thus, the patients included in this analysis had an accurate WD diagnosis, eliminating problems of misclassification in retrospective studies of databases. The results provide clinical information that may not be available from other sources. However, study generalizability may be limited because this cohort was diagnosed and managed by specialists in neurology, gastroenterology, and hepatology, which could differ from WD patients diagnosed and treated by general practitioners. It is also unclear how many patients had formal psychiatric assessments or neurological assessments; brain MRI was conducted in only about 12% of patients, whereas liver biopsy was performed in more than 60% of patients indicating a possible bias in favor of hepatic presentation assessment. A strength of this study is the documentation that US specialists with experience in WD do not follow published WD guidance recommendations regarding diagnosis and therapy. Of potential concern is the fact that the vast majority of patients in this study were White; which raises the prospect of bias based on racial disparity due to better access to specialist care by White, compared with either Black or Latino patients (19). The dominant disease presentations observed in this study may stem from difference in referral patterns to specialists, different conceptual approaches by clinicians to symptom clustering, and the heterogeneity of clinical WD, observed in this study and other studies. No QoL data were reported, as such data are not routinely captured in clinical practice.

### 4.2 Conclusions

This real-world study provides descriptive data regarding signs and symptoms of patients with WD diagnosed and managed by neurologists, psychiatrists, gastroenterologists and hepatologists in the United States. It documents that fact that only 40% of these clinicians with documented experience with WD patients follow published WD guidance recommendations. The range of patient symptoms reported in this analysis and in the literature highlights the complexity and heterogeneity of WD at presentation and during ∼1 year of follow up. This study also underscores the need for serial monitoring, laboratory testing, and access to multidisciplinary support. The associated neurologic, psychiatric, and hepatic signs, symptoms, and laboratory abnormalities of WD are a substantial burden for patients and a challenge for clinicians and society. Unmet needs include more comprehensive education for healthcare workers about WD and creation of tools to guide recognition of signs, symptoms, and laboratory test abnormalities that warrant an algorithmic approach to diagnosis and management.

## Data availability statement

The data analyzed in this study is subject to the following licenses/restrictions: Alexion, AstraZeneca Rare Disease will consider requests for disclosure of clinical study participant-level data provided that participant privacy is assured through methods like data de-identification, pseudonymization, or anonymization (as required by applicable law), and if such disclosure was included in the relevant study informed consent form or similar documentation. Qualified academic investigators may request participant-level clinical data and supporting documents (statistical analysis plan and protocol) pertaining to Alexion-sponsored studies. Further details regarding data availability and instructions for requesting information are available in the Alexion Clinical Trials Disclosure and Transparency Policy at https://alexion.com/our-research/research-and-development. Requests to access these datasets should be directed to Link to Data Request Form (https://alexion.com/contact-alexion/medical-information).

## Ethics statement

The studies involving humans were approved by New England Independent Review Board. The studies were conducted in accordance with the local legislation and institutional requirements. Written informed consent for participation was not required from the participants or the participants’ legal guardians/next of kin in accordance with the national legislation and institutional requirements.

## Author contributions

VM: Data curation, Formal analysis, Writing – review & editing. NK: Formal analysis, Writing – review & editing. JS: Formal analysis, Writing – review & editing. MK: Formal analysis, Writing – review & editing. JV: Formal analysis, Writing – review & editing.
